# Supplementary material for: Metallothionein Expression as a Physiological Response against Metal Toxicity in the Striped Rockcod Trematomus hansoni
Source: Int J Mol Sci. 2022 Oct 24;23(21):12799. doi: 10.3390/ijms232112799 (PMC9657541; doi:10.3390/ijms232112799)
Supplement: Supplementary file 1 [file ijms-23-12799-s001.zip › ijms-1977656-supplementary.pdf]

Table S1. Primer pairs used for qRT-PCR. Amplicon sizes and annealing temperatures (Ta) are also indicated.

PRIMER	Sequences 5′→3′	Amplicon (bp)	Ta (°C)
MT-1_fw	5′-GGACCTGCAACTGCGGAG-3′	123	60
MT-1_rv	5′-CTTTGCACACACAGCCAGAG-3′		60
MT-2_fw	5′-ACAACACGCTCTCATCTGA-3′	110	60
MT-2_rv	5′-AGTTGCAGGTCCCACTTTTG-3′		60
GAPDH_b_fw	5′-ATCACAGCCACACAGAAGAC-3′	126	60
GAPDH_b_rv	5′-AGGAATGACTTTGCCCACAG-3′		60
